# Advancing phylogenomics in Amaranthaceae sensu stricto: Development and application of a new nuclear target enrichment bait set

**DOI:** 10.1002/aps3.70019

**Published:** 2025-08-13

**Authors:** Tina Kiedaisch, Gudrun Kadereit, Anže Žerdoner Čalasan, Diego F. Morales‐Briones

**Affiliations:** ^1^ Prinzessin Therese von Bayern Chair for Systematics, Biodiversity and Evolution of Plants, Faculty of Biology Ludwig‐Maximilians‐Universität München Menzinger Straße 67 80638 Munich Germany; ^2^ Botanischer Garten München‐Nymphenburg (SNSB‐BGM), Staatliche Naturwissenschaftliche Sammlungen Bayerns Menzinger Straße 65 80638 Munich Germany; ^3^ Botanische Staatssammlung München (SNSB‐BSM), Staatliche Naturwissenschaftliche Sammlungen Bayerns Menzinger Straße 67 80638 Munich Germany; ^4^ Zentrum für Geobiologie und Biodiversitätsforschung an der Ludwig‐Maximilians‐Universität München Richard‐Wagner‐Str. 10 80333 Munich Germany

**Keywords:** Amaranthaceae, gene tree conflict, orthology inference, target enrichment, taxon‐specific bait set, whole‐genome duplications

## Abstract

**Premise:**

Current phylogenies of Amaranthaceae sensu stricto (s.s.) are inadequately sampled and resolved to reflect the entire evolutionary history of the lineage, which is likely complex due to at least three whole‐genome duplication events, occasionally followed by subsequent additional polyploidization events and rapid diversification of individual sublineages. We designed a new target enrichment bait set to overcome these challenges when reconstructing a phylogeny and demonstrated its applicability to the entire Amaranthaceae s.s. lineage.

**Methods:**

We analyzed 12,775 orthologous and low‐copy genes from a previous comprehensive transcriptomic study for marker selection. Following a newly developed approach that allows the selection of long exons and thus avoids the assembly of chimeric loci, we selected 1000 orthologous exons for phylogenomic analyses.

**Results:**

Our in vivo application showed a high locus recovery rate across all major clades of Amaranthaceae s.s., generated a robust phylogenetic tree, and clarified previously ambiguous relationships of the genera *Bosea* and *Charpentiera*. Gene tree conflict analysis revealed mainly high levels of gene tree concordance within the lineage, with a few notable exceptions.

**Discussion:**

The Amaranthaceae1000 kit will provide the basis for a phylogenetic tree across the Amaranthaceae s.s., facilitating future studies on systematics, diversification, and genome evolution within this economically important lineage.

The advent of next‐generation sequencing (NGS) technologies revolutionized the field of phylogenomics by enabling millions of individual sequencing reactions to be performed simultaneously, dramatically increasing throughput (Shendure and Ji, 2008). Although NGS made whole‐genome sequencing more affordable and efficient, it also introduced data analysis and storage challenges (Batley and Edwards, 2009). For phylogenomic studies, especially of plant groups with large and polyploid genomes, sequencing entire genomes often remains unfeasible, and focusing on a set of preselected genomic regions of interest is a more cost‐effective and convenient approach. Therefore, target‐enriched or hybridization‐based sequencing methods have become more popular in botanical research in recent decades (Mamanova et al., 2010). Another major advantage of target‐enriched approaches is their suitability for fragmented DNA, as they allow for the use of herbarium material, a valuable resource, especially for taxa that are difficult to collect because they are rare or grow in remote regions (Hale et al., 2020). While “universal” bait sets like Angiosperms353 have been widely applied in angiosperms (Johnson et al., 2019; Zuntini et al., 2024), numerous studies have illustrated their limitations (e.g., Lee et al., 2021; Yardeni et al., 2022; Haigh et al., 2023; Helmstetter et al., 2025) and underscored the need for more specific bait sets tailored to particular taxonomic groups. The level of taxonomic specificity varies, ranging from the family level (Nikolov et al., 2019; Christe et al., 2021; Eserman et al., 2021) to the genus or even species level (Bogarín et al., 2018; Villaverde et al., 2018).

Selecting loci for target enrichment is a crucial step in phylogenomic studies. Many studies aim to select single‐copy nuclear genes, such as ribosomal or housekeeping genes (Eserman et al., 2021; Acha and Majure, 2022; Timilsena et al., 2022), to avoid conflicts with paralogous copies in downstream analyses. However, gene duplications are widespread in plants, and many angiosperm families tend to retain duplicated genes that evolve independently (Li et al., 2016). In such cases, the history of these genes may not accurately reflect the species’ history, leading to either false or biased topologies. It is therefore essential to identify orthologous genes that are the product of speciation events and share a common ancestor. In contrast, paralogous genes arising from gene duplication events can introduce misleading signals into phylogenetic analyses (Fitch, 1970). Hence, high‐copy and paralogous loci should be excluded when selecting loci for bait design.

Orthology can be inferred using two main approaches: tree‐based methods and graph‐based methods. The tree‐based approach uses phylogenetic tree topologies to infer orthology from transcriptomic or genomic data (Gabaldón, 2008). In contrast, the graph‐based approach relies on pairwise sequence comparisons, typically performed using all‐against‐all BLAST searches. In doing so, orthology is inferred based on sequence similarity, as orthologs tend to have the highest sequence similarity between species (Gabaldón and Koonin, 2013). Similarity‐based approaches are the most commonly used in various pipelines for designing baits. MarkerMiner (Chamala et al., 2015), for example, performs a reciprocal BLAST search against a selected reference to identify putative orthologs. CAPTUS (Ortiz et al., 2024) employs a clustering approach using MMseqs2 (Many‐against‐Many sequence searching) (Steinegger and Söding, 2017). Other methods, such as Hyb‐Seq (Weitemier et al., 2014) and the approach described by Folk et al. (2015), compare putative orthologs to databases of known orthologs or single‐copy loci. These approaches limit the number of putative loci to those in the reference database, which may overlook lineage‐specific orthologs not included in the reference. Furthermore, sequence similarity–based methods can be prone to errors due to incomplete and heterogeneous datasets caused by lineage‐specific gene loss or duplication events, leading to misinterpretations (Koonin, 2005). Therefore, tree‐based methods are considered more reliable for determining orthologs, as they adhere strictly to the formal definition of orthology (Gabaldón, 2008).

Several tools for tree‐based orthology inference exist. Most pipelines first construct homolog gene trees and then detect subtrees with only one single sequence per taxon in a second step (Chiu et al., 2006; Dunn et al., 2013). A comparable tree‐based approach from Yang and Smith (2014), however, provides different algorithms to prune orthologous subtrees from homologous gene trees: maximum inclusion (MI), rooted ingroups (RT), and monophyletic outgroups (MO). The advantage of these methods is that they do not require a reference database and can therefore be applied to non‐model organisms (Yang and Smith, 2014).

Amaranthaceae sensu stricto (s.s.) is a well‐supported clade within the broader circumscription of Amaranthaceae sensu lato (s.l.) (Huang et al., 2020; Morales‐Briones et al., 2021; Xu et al., 2024). This group comprises approximately 900 species in 81 genera, which are further organized into five well‐supported monophyletic tribes: Achyranthoids, Aervoids, Amaranthoids, Celosioids, and Gomphrenoids (Hernández‐Ledesma et al., 2015; Hammer et al., 2019; Di Vincenzo et al., 2025). The origin of the family remains unknown, and a recent study unveiled that the five tribes probably originated simultaneously via a rapid radiation event (Morales‐Briones et al., 2021). *Bosea* L. and *Charpentiera* Gaudich., which have been placed as sister taxa to the rest of the clade (Müller and Borsch, 2005), have a widely disjunct distribution. *Bosea* can be found in Macaronesia, the eastern Mediterranean, and the Himalayas, while *Charpentiera* occurs in Hawaii and French Polynesia (Kadereit et al., 2003; Di Vincenzo et al., 2018). Previous phylogenies of Amaranthaceae s.s. relied mostly on a few plastid (*trnL‐F*, *rpl16, trnK*, *matK*) or nuclear loci (*ITS*, *A36*, *G3PDH*, *waxy*), and therefore displayed numerous poorly resolved relationships, especially on the genus level (Müller and Borsch, 2005; Sánchez‐Del Pino et al., 2009, 2012; Hammer et al., 2015; Bena et al., 2017; Waselkov et al., 2018; Limarino and Borsch, 2020; Bena et al., 2024). To date, there are only a few studies that are based on numerous loci; these include a plastome‐based phylogeny for the Australian genus *Ptilotus* R. Br. (Hammer et al., 2019) and two phylogenies encompassing the whole Amaranthaceae s.l. family (with an incomplete sampling of Amaranthaceae s.s.) based on plastome (Xu et al., 2024) and transcriptomic data (Morales‐Briones et al., 2021). Given the lack of a robust phylogenomic framework, combined with the important role of Amaranthaceae s.s. as both crop plants (Joshi and Verma, 2020; Aderibigbe et al., 2022) and noxious weeds (Bayón, 2022; Roberts and Florentine, 2022), there is a clear need for a deeper understanding of the evolution and systematics of this lineage.

The genetic complexity of Amaranthaceae s.s. presents significant challenges for phylogenomic studies. Genome sizes of taxa in the clade vary from 1 C = 0.48 pg (*Amaranthus palmeri* S. Watson; Bennett and Smith, 1976) to 1 C = 4.9 pg (*Celosia whitei* W. F. Grant; Nath et al., 1992), and the polyploidy level can reach up to 12× in *C. whitei* (Nath et al., 1992). Furthermore, three whole‐genome duplication (WGD) events have been identified in the backbone of the clade (Yang et al., 2018), adding to the complexity of its evolutionary history. Given that a target enrichment approach is particularly effective for taxa with large and/or polyploid genomes and has been successfully applied before (e.g., Morales‐Briones et al., 2021; Mendez‐Reneau et al., 2023; Walden et al., 2024), we employed the same method for Amaranthaceae s.s.

Here, we present a new workflow for the design of a taxon‐specific bait set for Amaranthaceae s.s., called Amaranthaceae1000. This workflow utilizes a tree‐based orthology inference approach, ensuring accurate identification of orthologous loci without relying on reference databases. In addition, our method prioritizes the selection of long loci, reducing the risk of chimeric gene assembly. We show that these newly developed nuclear markers enable a lineage‐wide application, successfully overcoming the technical challenges posed by the genomic complexity of Amaranthaceae s.s. With this approach, we aim to provide a robust phylogenomic framework that will serve as a prerequisite to gain deeper insights into the spatial and temporal evolution of this economically and ecologically important lineage.

## METHODS

### Selection of target loci

We extracted 12,775 FASTA files from the ortholog gene trees generated by Morales‐Briones et al. (2021) and filtered out all but the Amaranthaceae s.s. sequences. The orthologs were inferred using the tree‐based MO approach developed by Yang and Smith (2014). To identify exon–intron boundaries, we downloaded the *Amaranthus hypochondriacus* L. v2.1 genome available on Phytozome v13 (Goodstein et al., 2012). We then masked the introns by extracting all genes and exons from the annotated genome and subtracted them from their respective genes using BEDTools v2.30.0 (Quinlan and Hall, 2010). From the 12,775 orthologs, we proceeded with a subset of 10,612 orthologs that comprised the sequence of *A. hypochondriacus*. We then aligned the 10,612 orthologs of the Amaranthaceae s.s. taxa using MAFFT v7.490 (Katoh and Standley, 2013) and, in a second step, added the intron‐masked genome of *A. hypochondriacus* into the existing alignment using the option ‐‐add (Katoh and Frith, 2012).

Exons were split under two conditions: (i) if the intron was present in the genomic reference and (ii) if gaps were observed in the transcriptomes. These criteria ensured the retrieval of complete exons, resulting in 73,262 exons with an average sequence length of 285.67 bp. The scripts used for this process are available at https://github.com/tinakiedaisch/bait_design_from_orthologs (see Data Availability Statement). The exons were filtered by length, and 6168 exons longer than 700 bp were retained. Next, we aimed to remove highly conserved loci by targeting exons with more than 2% parsimony informative sites. Informativeness was assessed using PhyKIT (Steenwyk et al., 2021), resulting in the retention of 5947 exons that exceeded this threshold. Finally, sequences with a gap proportion greater than 0.5 were identified using the get_sequences_gaps_ratio.py script of trimAL v.3.29 (Capella‐Gutiérrez et al., 2009) and removed from the alignments.

We realigned the exons using the OMM_MACSE v12.01 pipeline (Ranwez et al., 2021) to preserve their amino acid translation, remove non‐homologous sequence fragments, and trim extremities. The resulting alignments were again filtered with trimAL under the conditions mentioned above. The remaining exons were filtered in Geneious Prime 2023.2.1 (Biomatters Ltd., Auckland, New Zealand; http://www.geneious.com/), retaining only those with a minimum length of 800 bp, a minimum pairwise identity of 75%, and present in at least 10 sequences per alignment. After the filtering, we manually reviewed the 1894 exons and removed those with excessive gaps and stop codons, resulting in 1216 carefully selected exons for downstream analyses. We ensured that all loci included representatives from both major clades (clade 1: Aervoids, Achyranthoids, and Gomphrenoids; clade 2: Celosioids and Amaranthoids) to guarantee lineage‐wide application of the baits.

Because three WGD events in Amaranthaceae s.s. have been detected (Yang et al., 2018), finding single‐copy loci was unrealistic. To get a general impression of the duplication rates, we thus counted the occurrence of individual taxa in each of the 14,549 isoform‐masked homologous gene trees, which were accessed through the supplementary material of Morales‐Briones et al. (2021).

*Deeringia amaranthoides* (Lam.) Merr. and *Hermbstaedtia glauca* (J. C. Wendl.) Rchb. ex Steud. did not undergo a WGD (Morales‐Briones, unpublished work) and were therefore used to identify whether the selected loci belonged to large gene families. Loci with more than three duplications in either species were removed, leaving 1200 exons (in 1163 genes) for the final step of the bait design. We randomly selected 1000 exons to comply with the size restrictions of the 40,000‐bait kit. Summary statistics of the selected exons were performed with AMAS (Borowiec, 2017).

Finally, we wanted to determine whether one sequence (representing either clade 1 with Gomphrenoids, Achyranthoids, and Aervoids or clade 2 with Amaranthoids and Celosioids) or two sequences (representing both clades 1 and 2) should be selected for bait design. To assess how the choice of representative sequences could potentially affect the success of locus recovery, we performed an in‐silico capture analysis using CAPTUS v1.0.1 (Ortiz et al., 2024). For this, we generated three different target files: (i) containing the targeted 1000 loci from species of clade 1 (see above), (ii) containing the targeted 1000 loci from species of clade 2 (see above), and (iii) containing the targeted 1000 loci from species of both clades 1 and 2 representing all major groups in Amaranthaceae s.s. These three target files were used for exon extraction from the 29 Amaranthaceae s.s. transcriptomes of Morales‐Briones et al. (2021). We observed that target files constructed following strategies i and ii resulted in a biased capture efficiency, with reduced efficiency in the clade absent from the target file. In contrast, the target file following strategy iii resulted in equally high capture efficiency in both clades. We therefore followed strategy iii by randomly selecting two sequences with the fewest gaps in each clade for the bait design. Bait synthesis was performed using myBaits at Daicel Arbor BioSciences (Ann Arbor, Michigan, USA). A total of 39,091 biotinylated 120‐nucleotide RNA probes were designed with a 2× tiling strategy.

### Taxon sampling

To demonstrate the functionality of the designed baits in vitro, we selected 24 species from Amaranthaceae s.s. (Appendix 1), covering Achyranthoids, Aervoids, Amaranthoids, Celosioids, and Gomphrenoids (Hernández‐Ledesma et al., 2015), as well as *Bosea* and *Charpentiera*. The samples were collected from the herbaria CANB, M, MSB, and PERTH (herbarium acronyms per Index Herbariorum [Thiers, 2025]).

From the original Amaranthaceae s.l. dataset of Morales‐Briones et al. (2021), we kept the following 16 samples for the phylogenetic reconstruction to represent the eight major clades: *Suaeda divaricata* Moq. and *Suaeda maritima* (L.) Dumort. from the Suaedoideae; *Salicornia pacifica* Standl. and *Tecticornia pergranulata* (J. M. Black) K. A. Sheph. & Paul G. Wilson from the Salicornioideae; *Haloxylon ammodendron* (C. A. Mey.) Bunge ex Fenzl and *Kali collinum* (Pall.) Akhani & Roalson (syn. *Salsola collina* Pall.) from the Salsoloideae; *Bassia scoparia* (L.) A. J. Scott and *Eokochia saxicola* (Guss.) Freitag & G. Kadereit from the Camphorosmoideae; *Chenopodium quinoa* Willd. and *C. amaranticolor* H. J. Coste & A. Reyn. from the Chenopodioideae; *Agriophyllum squarrosum* (L.) Moq. and *Corispermum hyssopifolium* L. from the Corispermoideae; *Beta macrocarpa* Guss. and *Hablitzia tamnoides* M. Bieb. from the Betoideae; and *Nitrophila occidentalis* (Moq.) S. Watson and *Polycnemum majus* A. Braun ex Bogenh. from the Polycnemoideae. In addition, we included 29 transcriptomes of Amaranthaceae s.s., also derived from Morales‐Briones et al. (2021), to assess the accuracy of our sample placements within the phylogenetic framework.

Moreover, transcriptomes from two Achatocarpaceae species, *Phaulothamnus spinescens* A. Gray and *Achatocarpus gracilis* H. Walter, which are sister to Amaranthaceae s.l. (Morales‐Briones et al., 2021), along with those of the more distantly related *Spergularia media* (L.) C. Presl, *Dianthus caryophyllus* L., *Illecebrum verticillatum* L., *Herniaria latifolia* Lapeyr., *Corrigiola litoralis* L., *Mesembryanthemum crystallinum* L., *Delosperma echinatum* (Lam.) Schwantes, *Commicarpus scandens* (L.) Standl., *Mollugo pentaphylla* L., and *Microtea debilis* Sw. from the Caryophyllales were chosen as outgroups.

### DNA extraction, library preparation, and sequencing

DNA was extracted from 20–30 mg of leaf material using the DNeasy Plant Mini Kit (QIAGEN, Hilden, Germany) and eluted in 75 µL of 10 mM Tris. DNA quantity was determined with a high‐sensitivity assay kit using the Qubit 4 Fluorometer (Thermo Fisher Scientific, Waltham, Massachusetts, USA), and fragment size was assessed visually via electrophoresis on a 0.8% agarose gel. For long DNA fragments (>1000 bp), 25 µL was sheared to an average size of 350 bp with an M220 Focused‐ultrasonicator with the M220 Holder XTU Insert microTUBE 50 µL (Covaris, Woburn, Massachusetts, USA) prior to library preparation.

One hundred nanograms of DNA was aliquoted in 25 µL of 10 mM Tris and used directly for library preparation. We used the NEBNext Ultra II Kit for Illumina (New England Biolabs, Ipswich, Massachusetts, USA) following the manufacturer's protocol (for half‐volume reactions). After the adapter ligation step, size selection was performed for samples with fragment sizes of 500–1000 bp. The DNA quantity was then measured with the Qubit 4 Fluorometer, and fragment size was assessed with the 4150 TapeStation System using the High Sensitivity D1000 ScreenTape Assay (Agilent Technologies, Santa Clara, California, USA). All libraries were dual‐indexed using NEBNext Multiplex Oligos for Illumina (NEB #E6444, New England Biolabs).

Finished libraries were normalized to 10 nM, and up to 24 samples of similar sizes were pooled together, with the final pool containing 623.99 ng of DNA in 240 µL. The DNA was concentrated to a final volume of 7 µL ddH_2_O using a vacuum pump. The hybridization followed the myBaits Hybridization Capture for Targeted NGS protocol version 5.02 (Daicel Arbor Biosciences) using the Q5 polymerase. The hybridization took place for 17 h at 62°C. Captured DNA was enriched with a 12‐cycle PCR and a bead clean‐up at a 0.9× ratio. The final DNA concentration and size were measured with the High Sensitivity D1000 TapeStation (Agilent Technologies). Sequencing was carried out at Novogene (Munich, Germany), with a targeted amount of 2.3 Gbp per sample, resulting in 900× coverage of the 2,571,494 bp total reference length.

### Sequence assembly

Read quality was assessed with FastQC v0.11.7 (Andrews, 2010), and results were compiled with MultiQC v1.22 (Ewels et al., 2016). We deduplicated the reads using ParDRe v2.2.5 (González‐Domínguez and Schmidt, 2016) and used Trimmomatic v0.39 (Bolger et al., 2014) with the parameters SLIDINGWINDOW:4:5 LEADING:5 TRAILING:5 MINLEN:25 to remove sequencing adapters and low‐quality reads. For the in silico capture in HybPiper2 v2.1.6, we chose BLASTX as a mapping tool (Johnson et al., 2016). For loci extraction, we created two separate target files, each representing one of the two large clades in Amaranthaceae (Clade 1: Aervoids, Achyranthoids, Gomphrenoids; Clade 2: Celosioids, Amaranthoids). The similarity threshold to map the reads was adjusted for each sample according to its proximity to the species in the reference file (‐‐thresh option) and ranged between 75% and 85%. An individual value (‐‐cov_cutoff option) in SPAdes (Prjibelski et al., 2020) was used to adjust the coverage during loci assembly for each sample. The coverage was calculated based on a previous HybPiper run under default coverage settings using a custom R script. This custom assembly ensured a higher quality of the extracted loci and fewer misassemblies.

For de novo chloroplast assembly, we applied Fast‐Plast v1.2.6 (McKain, 2017) using the untrimmed, de‐duplicated reads. As a reference, we used the whole chloroplast genomes of all members of the order Caryophyllales integrated in the program.

### Orthology inference and phylogenomic analysis

To include the transcriptomic data of Morales‐Briones et al. (2021), we split the 1000 targeted exons from the orthologous genes described above. Therefore, all major clades of Amaranthaceae s.l., representatives of Amaranthaceae s.s., and their respective outgroups from the Caryophyllales were incorporated.

We inferred orthology from the paralog_no_chimeras output from HybPiper2 following the phylogenomic dataset construction pipeline (Yang and Smith, 2014) with modifications from Morales‐Briones et al. (2021). Because we had included a comprehensive list of outgroup taxa, we followed the MO approach with at least 15 ingroup taxa (out of 53 total) (Yang and Smith, 2014). The individual 972 inferred orthologous loci were aligned using MACSE v2.07 (Ranwez et al., 2018), and columns with more than 70% missing data were trimmed using pxclsq from phyx v.1.3 (Brown et al., 2017). Gene trees were inferred using a maximum likelihood (ML) approach in IQ‐TREE2 v2.3.1 (Minh et al., 2020) with an ultrafast bootstrap approximation (UFBoot) of 1000 (Hoang et al., 2018). Branches with UFBoot support values below 70 were collapsed. The species tree was reconstructed under a coalescent‐based model with ASTRAL IV v1.20.4.6 (Zhang and Mirarab, 2022a) using the ML gene trees. Node support was expressed in local posterior probability values (LPP). In addition, we employed Astral‐Pro3 (ASTRAL for PaRalogs and Orthologs) v1.20.3.6 (Zhang and Mirarab, 2022b) to reconstruct a species tree using all cleaned homologous trees, as it allows for multi‐copy genes.

### Gene tree discordance analysis

For gene tree discordance analysis, we used PhyParts v0.0.1 (Smith et al., 2015), a tool that classifies the nodes of gene trees into four categories: (i) supporting the species tree topology, (ii) supporting the main alternative topology, (iii) supporting all other alternative topologies, and (iv) uninformative (including missing data). The conflict can be quantified by calculating each node's internode certainty all (ICA) values. An ICA close to 1 indicates strong concordance, close to 0 indicates equal support for one or more conflicting bipartitions, and a negative ICA indicates discordance.

We rooted the uncollapsed gene trees and the species tree on the outgroups consisting of *Commicarpus scandens*, *Corrigiola litoralis*, *Delosperma echinatum*, *Dianthus caryophyllus*, *Herniaria latifolia*, *Illecebrum verticillatum*, *Mesembryanthemum crystallinum*, *Microtea debilis*, *Mollugo pentaphylla*, and *Spergularia media* (all Caryophyllales). Because each node could have a different number of gene trees, which may skew the proportion of uninformative gene trees, the PhyParts results were plotted, including informativeness and missingness. The node was treated as uninformative if the UFBoot was <70%. We plotted the results using the Python script available at phypartspiecharts_missing_uninformative.py (https://bitbucket.org/dfmoralesb/target_enrichment_orthology/src/master/).

To further test for confidence, consistency, and informativeness of the phylogeny, we used Quartet Sampling v1.3.1 (QS) (Pease et al., 2018). By resampling the quartet tree counts, three scores were calculated for each internal branch of the phylogeny: (i) the Quartet Concordance (QC) score, which is similar to ICA and quantifies the concordant quartet; (ii) the Quartet Differential (QD) score, which measures the disparity between the sampled proportions of the two discordant topologies and can therefore indicate when one alternative relationship is sampled more frequently than the other; and (iii) the Quartet Informativeness (QI) score, which quantifies the proportion of informative replicates.

As input, we concatenated the trimmed orthologous sequences with AMAS (Borowiec, 2017) and converted them from FASTA to PHYLIP format using pxs2phy from phyx. Before running the QS analysis using IQ‐TREE with 1000 replicates, we rerooted the species tree using pxrr from phyx.

### Mapping whole‐genome duplications

Lastly, we wanted to explore whether the already known WGD in Amaranthaceae s.s. (Yang et al., 2018) could be detected in our target‐enriched dataset. To exclude the potential bias from the transcriptomic data, we performed this analysis only on the 24 newly sequenced samples enriched with the Amaranthaceae1000 custom baits, using available scripts from Yang et al. (2018) (https://bitbucket.org/blackrim/clustering).

We extracted the rooted orthogroups (ingroup lineages with genes descended from a single ancestor) from our cleaned homologous trees, setting the MIN_INGROUP_TAXA feature to 10 (extract_clades.py). We then mapped the duplications from each orthogroup onto the species tree (map_dups_concordant.py) and plotted the calculated duplication percentages onto the individual branches (plot_branch_labels.py).

## RESULTS

### Bait design

**Figure 1:**
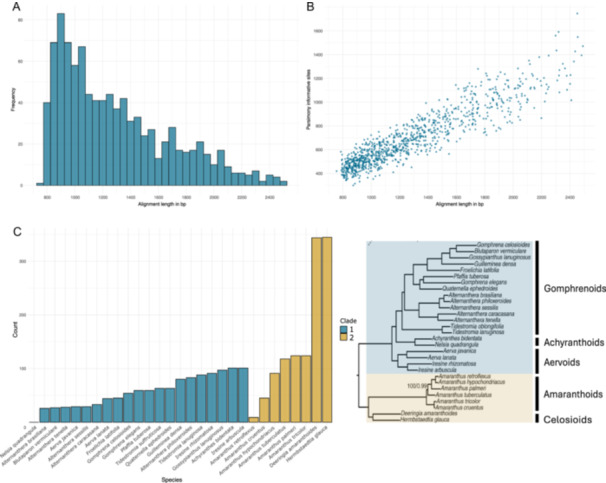
Summary statistics for 1000 loci selected for bait design. (A) Length distribution of the included loci (in base pairs). (B) Scatterplot showing the relationship between locus length (in base pairs) and the number of parsimony‐informative sites. (C) Frequency of species sequence representation per clade in the bait design for the 1000 loci.

The final bait set included 1000 exons from 989 genes and targeted 1,294,760 bp. A minimum of 10 taxa (out of 29 taxa) were represented in all loci. The length of the targeted loci ranged between 800 and 2500 bp, with an average locus length of 1295 bp (Figure 1A). The proportion of parsimony informative sites ranged between 0.21 and 0.45 (average 0.31) and correlated with sequence length (Figure 1B).

The in‐silico capture in CAPTUS was used to determine whether one or two sequences should be used for bait design, and it showed that two sequences resulted in better locus capture across the entire Amaranthaceae s.s. When only sequences from either clade were used, we observed reduced capture efficiency in the non‐sampled clade (Appendix S1, see Supporting Information with this article). Therefore, for each locus, one sequence with the fewest gaps from each clade was randomly selected for bait design (Figure 1C). Here, we should briefly mention that the incomplete and fragmented transcriptome of *Nelsia quadrangula* (Engl.) Schinz was never selected due to its low quality and poor assembly, but all 28 other species are represented in the sequences for bait design (Figure 1C). Most represented are *Deeringia amaranthoides* and *Hermbstaedtia glauca* from the Celosioids, which were newly generated in Morales‐Briones et al. (2021) and had good‐quality transcriptomes, while *Amaranthus retroflexus* L. was less represented due to its incomplete transcriptome (Figure 1C).

### Sequence data and orthology inference

**Figure 2:**
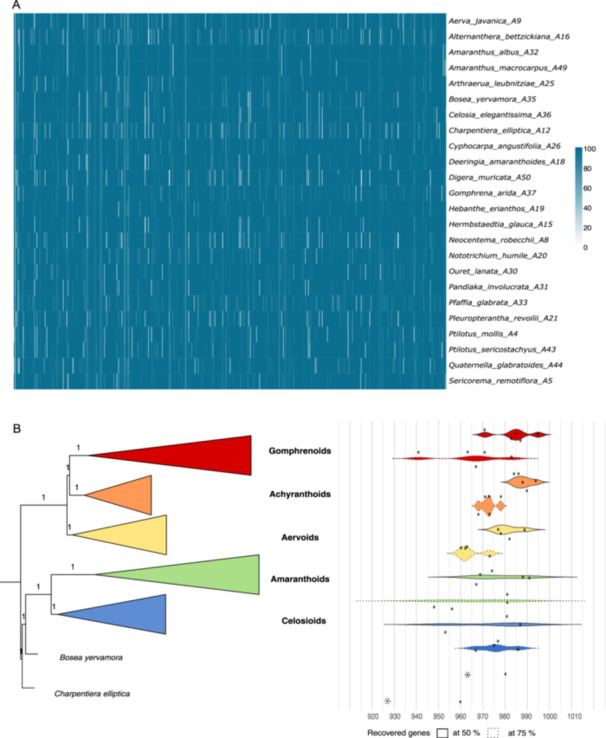
Locus recovery success. (A) Percentage of the reference length recovered for 24 species of Amaranthaceae s.s. for 1000 loci, ranging from 0–100%. (B) Number of recovered genes reaching 50% (solid line) and 75% (dotted line) of the reference length across the five major clades of Amaranthaceae s.s., as well as the two genera *Bosea* and *Charpentiera*.

We generated 3.8 to 13 million raw reads per sample, about half of which consisted of PCR duplicates (on average). Between 1.2 and 5.7 million reads were mapped to the target file (9.4–49.2%; average: 36.6% of reads on target). Between 960 and 995 genes with more than 50% of the targeted locus length (average: 982 genes, 98.2%) and between 927 and 983 recovered genes with more than 75% of the targeted locus length (average: 966 genes, 96.6%) were recovered (Figure 2). All 1000 loci contained sequences for which more than 50% of the length was recovered, with only one locus (AH017606_E2) recovered for less than 50% of the length in any of the sampled species (Figure 2A). All clades of Amaranthaceae s.s. showed comparable recovery efficiency at both 50% and 75% of the target locus length: Achyranthoids (988 and 973 genes), Aervoids (982 and 965 genes), Amaranthoids (982 and 963 genes), Celosioids (984 and 973 genes), and Gomphrenoids (985 and 965 genes), respectively (Figure 2B). In addition to the nuclear data, the percentage of chloroplast genes recovered ranged from 3.7% (*Arthraerua leubnitziae* (Kuntze) Schinz) to 82.7% (*Ptilotus mollis* Benl), with an average of 43.4% (Appendix S2).

Out of 972 pruned orthologs, between 606 (in *Ptilotus mollis*) and 937 (in *Bosea yervamora* L.) were retrieved per sample (with an average of 729). Trimmed MO orthologs ranged from 756 to 2490 characters (with an average of 1290) and about 2.7% missing data (from 0% to 32%).

### Phylogenetic reconstruction

**Figure 3:**
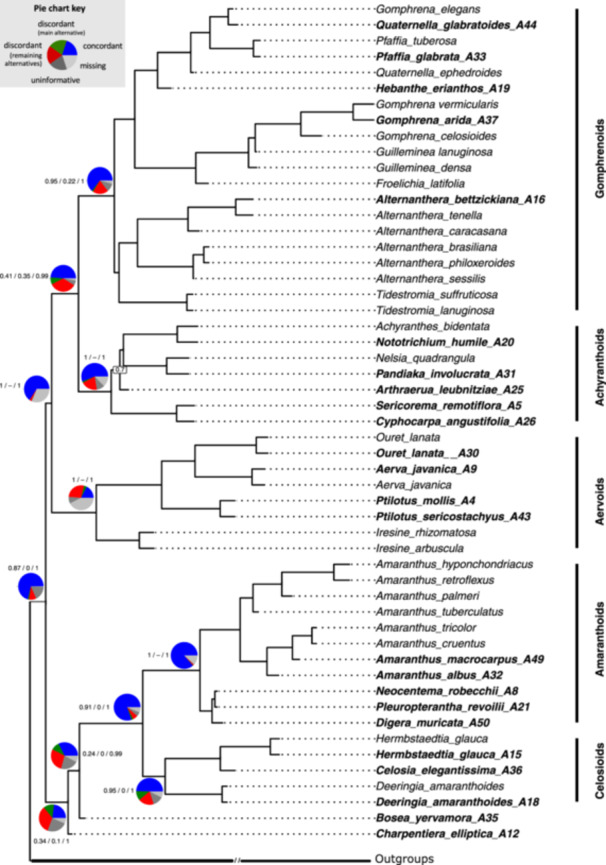
Coalescent phylogenetic inference based on 57 transcriptomes and 24 samples sequenced with the Amaranthaceae1000 baits. The species tree resulted from an ASTRAL IV analysis of “monophyletic outgroup” (MO) ortholog trees. Species sequenced with the bait set are in bold, followed by their respective laboratory accession numbers. All branches have maximum support, except those marked with an asterisk, with local posterior probabilities (LPP) of 0.9 and 0.98 (from top to bottom). The tree is rooted in members of the Caryophyllales. Pie charts represent results from a PhyParts analysis, with blue indicating concordance, red indicating discordance, green showing the main alternative topology, dark gray representing informativeness, and light gray denoting missing data. Branch values correspond to quartet sampling analysis, showing the quartet concordance (QC) score, quartet differential (QD) score, and quartet informativeness (QI) score, respectively.

The coalescent ASTRAL tree confirmed the monophyly of Amaranthaceae s.s. within Amaranthaceae s.l. with the former Chenopodiaceae, Betoideae, and Polycnemoideae (LPP = 1.00; Figure 3). Within Amaranthaceae s.s., we recovered five clades, and the relationships between them were resolved with maximum support (LPP = 1.00; Figure 3). These five clades clustered into two major lineages: one containing the Gomphrenoids, the Achyranthoids, and the Aervoids, and the other containing the Amaranthoids and the Celosioids, with the genera *Bosea* and *Charpentiera* forming a sister grade to the latter major lineage. The placement of the 24 newly sequenced samples with the Amaranthaceae1000 baits was consistent with the phylogenetic framework of the transcriptomic data (Figure 3). The genera *Quaternella* Pedersen and *Gomphrena* L. were recovered as non‐monophyletic, whereas all other genera, albeit not densely sampled, were found to be monophyletic. The newly sequenced Australian species *Gomphrena arida* J. Palmer was nested within a well‐supported clade (LPP = 1.00) comprising *G. vermicularis* L. (syn. *Blutaparon vermiculare* (L.) Mears), *G. arida*, *G. celosioides* Mart., and *Gossypianthus lanuginosus* (Poir.) Moq. (syn. *Gomphrena lanuparonychioides* T. Ortuño & Borsch). For the first time, the genus *Neocentema* Schinz could be placed phylogenetically within the Amaranthoids with maximum support (LPP = 1; Figure 3). All results were congruent with the Astral‐Pro3 inference that was performed on all homologs (Appendix S3).

### Gene tree discordance

The Gomphrenoids and the Achyranthoids showed a high degree of gene tree concordance (ICA = 0.61 and ICA = 0.50, respectively) and a strong QS score (0.95/0.22/1 and 1/–/1, respectively). However, their sister group relationship remained controversial with moderate discordance (ICA = 0.29, QS score 0.41/0.35/0.99), indicating a main alternative topology. The Aervoids had the maximum QS score but suffered from a high level of missing data (ICA = 0.26), whereas the Amaranthoids had high levels of gene tree concordance (ICA = 0.89) and a maximum QS score. The Celosioids showed some gene tree discordance but had a good QS score (ICA = 0.63, QS score 0.95/0/1). The highest levels of gene tree discordance in Amaranthaceae s.s. were found at the base of Amaranthaceae s.s., with *Bosea* placed as sister to the Amaranthoids and Celosioids, and *Charpentiera* recovered as sister to both (QS score 0.34/0.1/1 and −0.24/0/0.99, respectively). The analysis yielded a main alternative at both nodes, with *Bosea* and *Charpentiera* resolved as sister clades. Nevertheless, the relationship between the Amaranthoids and the Celosioids was highly congruent, as was the monophyly of the two clades.

### Whole‐genome duplications

We detected the highest percentage of duplicated genes at the base of clade 1 (Gomphrenoids, Achyranthoids, and Aervoids) with 37.79% (Appendix S4). On the subsequent branches, the proportion of duplicated genes was still elevated, with 14.77% for the Achyranthoids and Gomphrenoids and 10.49% for the Aervoids. A second WGD event was detected at the base of the Amaranthoids with 8.52% duplicated genes. The known WGD event in *Alternanthera* Forssk. was undetected (Yang et al., 2018).

## DISCUSSION

Target enrichment strategies have transformed molecular systematics (Soltis et al., 2013), and the subsequent development of “universal” angiosperm baits (Johnson et al., 2019) has played an important role in resolving the phylogenies of many taxa (Antonelli et al., 2021; Thomas et al., 2021; Giaretta et al., 2022). This success has contributed immensely to the angiosperm tree of life, which includes over 8000 genera and demonstrates the potential of universal baits (Zuntini et al., 2024). However, the relationship cannot be disentangled for complex groups using this universal bait set alone (Lee et al., 2021). To address this drawback, custom baits have become increasingly popular in recent years and have been shown to function at different taxonomic scales (Bogarín et al., 2018; Villaverde et al., 2018; Nikolov et al., 2019; Christe et al., 2021; Eserman et al., 2021). Here, we introduce the Amaranthaceae1000 bait kit, which retrieves up to 1000 low‐copy orthologous nuclear exons for phylogenomic analyses of Amaranthaceae s.s. We show that almost all (98.2%) of the targeted genes can be recovered across Amaranthaceae s.s. and a robust phylogenetic framework can be established.

### Marker selection and capture efficiency

The efficiency of the probe set depends highly on the evolutionary distance between the taxa used for bait design and the studied taxa in question (Carlsen et al., 2018; Andermann et al., 2020; Veltman et al., 2024). Thereby, closely related taxa are expected to perform better, and the bait efficiency decreases as sequence similarity decreases in other, less closely related samples. For this reason, we used representatives of all major Amaranthaceae s.s. clades to design the baits (Figure 1C). We observed no difference in the captured genes between the five subclades (Figure 2B), concluding that the bait kit is suitable across the whole lineage. While not explicitly tested, our taxon sampling illustrates that the baits may have enough resolution power to resolve even lower generic levels, as with *Amaranthus* L. (Figure 3). However, denser sampling is needed to corroborate this claim, and further studies are currently underway in our lab.

Another important factor to consider when selecting markers for target enrichment is their length. Longer loci tend to contain more single‐nucleotide polymorphisms (SNPs) (e.g., Figure 1B), making them more informative for phylogenomic studies. However, it is crucial to recognize that in a species tree inference based on gene trees, each locus contributes one gene tree, regardless of its length. This means that while longer loci may capture more genetic variation, they require more sequencing resources but ultimately carry the same weight in downstream analyses as shorter loci. Nevertheless, shorter loci tend to have more gene tree errors (Zhang et al., 2017), affecting two‐step species tree inference methods like ASTRAL (Mirarab and Warnow, 2015). Furthermore, erroneous gene trees can affect orthology inference and downstream analyses (Morales‐Briones et al., 2022). For this reason, we targeted longer loci between 800 and 2500 bp (with an average length of 1295 bp) (Figure 1A) to provide sufficient SNP density without unnecessarily increasing sequencing costs or data complexity.

In the context of genome duplications, the risk of assembling chimeric genes increases, especially when selected markers include multi‐exon genes (Morales‐Briones et al., 2018). Such errors may obscure the evolutionary history of the locus in question, making orthology inference difficult. For instance, the universal Angiosperms353 bait set contains 2304 exons from 353 genes, with an average exon length of 165 bp (Johnson et al., 2019). While this bait set has proven effective in many applications, its use in polyploid species carries the risk of assembling genes containing exons from different gene copies. To address this issue, we prioritized the selection of longer exons (Figure 1A) to minimize the potential for compound loci and improve the reliability of orthology inference, as suggested in Morales‐Briones et al. (2022).

We found a relatively low percentage of on‐target reads (36.6%) compared with other custom baits: 42% in Zingiberaceae (Carlsen et al., 2018), 42.47% in *Arachis* L. (Peng et al., 2017), 48.6% in *Euphorbia* L. (Villaverde et al., 2018), 52% in Ochnaceae (Shah et al., 2021), and 73% in Bignoniaceae (Fonseca et al., 2023). However, these differences are strongly influenced by the choice of mapping tool. BLASTX, which we used, typically maps fewer reads than alignment‐based tools like BWA, because it performs protein‐level searches with stringent statistical filtering. In turn, BLASTX typically retrieves more genes, despite being computationally more demanding (https://github.com/mossmatters/HybPiper/wiki/Troubleshooting,-common-issues,-and-recommendations) (e.g., Maurin et al., 2021). This is highlighted in our data, where we show that, even if a low percentage of reads are mapped to the target (e.g., *Deeringia amaranthoides* A18: 9.4% on target), most of the loci are recovered (e.g., *Deeringia amaranthoides* A18: 975 genes at 75% length) (Appendix S5). Furthermore, we set strict requirements for locus assembly. Using a customized coverage cutoff for SPAdes (‐‐cov_cutoff 15 to 60) or the high percent identity threshold for retaining exon hits, we avoid assembling potential contaminations or chimeric exons consisting of contigs with very different coverage values.

### Paralog handling

While many bait design studies aim to select single‐copy genes to minimize downstream conflict with paralogs, we selected genes from tree‐inferred orthologs. Single‐copy loci often represent highly conserved genes involved in essential biological processes such as photosynthesis or the cell cycle, which often limits their use when addressing other evolutionary questions (De Smet et al., 2013; Li et al., 2016). In contrast, using non‐housekeeping orthologous genes allows us to potentially broaden the scope of the bait kit by targeting more variable genes that can be used to infer the evolutionary histories of lower taxonomic units. By directly using the orthologs for bait design, we ensure that we capture the copies that reflect the evolutionary history of the species in question. Nevertheless, despite numerous bioinformatic tools (Emms and Kelly, 2019; Grau‐Bové and Sebé‐Pedrós, 2021; Persson and Sonnhammer, 2022), determining orthology remains challenging, as duplicated regions may undergo re‐diploidization over time, resulting in hidden paralogy (Xiang et al., 2017; Bomblies, 2020).

Paralogs have gained attention in phylogenomic studies due to their potential to provide valuable evolutionary insights (Gardner et al., 2021; Morales‐Briones et al., 2022; Walden et al., 2024). In our study, we retained all paralogous copies extracted with HybPiper2 and inferred orthology using an automated tree‐based approach (Yang and Smith, 2014) rather than discarding paralogs at the outset and thus losing potentially important information (Ufimov et al., 2021). This method was developed for genomic or transcriptomic datasets but has also proven effective for paralogous loci from target enrichment datasets (Morales‐Briones et al., 2022). Using the MO approach, we retained 972 out of 1000 loci, while 853 genes were paralog‐flagged after extraction with HybPiper2 and, therefore, theoretically had to be excluded (Appendix S5). Hence, we concur with Ufimov et al. (2022) and Morales‐Briones et al. (2022) that, in the case of WGD, paralogous flagged loci should not be removed from the analysis as this will result in substantial data loss. Furthermore, by discarding paralogous flagged loci at the outset, valuable information about biological processes such as hybridization is lost (Joyce et al., 2025). Instead, all copies should be retrieved and orthologous alignments should be generated (Yang and Smith, 2014).

A notable drawback of the MO approach is its potential to introduce bias in cases of allopolyploidy (Morales‐Briones et al., 2022). Yang et al. (2018) identified two allopolyploidy events (AMAR1 and AMAR2) within Amaranthaceae s.s.: one at the base of the Aervoids and Gomphrenoids, and another within the Aervoids, between the species *Aerva javanica* (Burm. f.) Juss. ex Schult. and *Ouret lanata* (L.) Kuntze. These two nodes exhibit a high percentage of missing data in PhyParts (Figure 3), likely reflecting these challenges associated with allopolyploidy. In such cases, the MO algorithm can create imbalances between subtrees. This occurs because the algorithm prunes the subtree with fewer taxa, which may disproportionately affect one subgenome due to unequal gene loss following allopolyploidization. Additionally, the design of baits on allopolyploid taxa can introduce bias by preferentially targeting one subgenome over the other (Morales‐Briones et al., 2022). To address the heterogeneity of gene trees due to gene duplication and loss, other tree‐based orthology inference methods, such as the RT algorithm that keeps all subtrees (Yang and Smith, 2014), DISCO (Willson et al., 2022), or ASTRAL‐Pro (Zhang and Mirarab, 2022b), can be used instead (Appendix S3).

### Phylogenomics of Amaranthaceae s.s

The phylogenomic tree of Amaranthaceae s.s. was well‐supported and overall in line with existing phylogenies (Figure 3) (Müller and Borsch, 2005; Sánchez‐Del Pino et al., 2009, 2012; Hammer et al., 2015, 2019; Bena et al., 2017, 2024; Di Vincenzo et al., 2018, 2025; Waselkov et al., 2018; Huang et al., 2020; Limarino and Borsch, 2020; Morales‐Briones et al., 2021; Xu et al., 2024). We confirmed the topology among the five main clades—Gomphrenoids, Achyranthoids, Aervoids, Amaranthoids, and Celosioids. While many studies placed the genera *Bosea* and *Charpentiera* as a sister grade to the rest of Amaranthaceae s.s. (Kadereit et al., 2003; Müller and Borsch, 2005; Sage et al., 2007; Ogundipe and Chase, 2009; Bena et al., 2017; Di Vincenzo et al., 2018; Huang et al., 2020), we were able to resolve this relationship for the first time by using an NGS‐based approach and found that they are sister genera to the Amaranthoids and Celosioids. However, we discovered high levels of gene tree discordance between them (Figure 3), with the main alternative being that both lineages are sisters to each other (data not shown). In the past, the two genera *Bosea* and *Charpentiera* had been placed together with the genera *Amaranthus* and *Chamissoa* Kunth in the subtribe Amaranthineae of Amarantheae (Townsend, 1993), and proximity to *Deeringia* R. Br. had also been suggested (Kadereit et al., 2003). Although both claims are based on limited morphology only, they are substantiated by our results.

Within the Gomphrenoids, the genus *Alternanthera* was found to be monophyletic and sister to *Tidestromia* Standl., as found in previous studies (Sánchez‐Del Pino et al., 2012; Bena et al., 2017; Morales‐Briones et al., 2021). In contrast, the genera *Gomphrena* and *Quaternella* were retrieved as polyphyletic. While the genus *Quaternella* has never been included in a phylogenetic reconstruction, except for one species (*Quaternella ephedroides* Pedersen) (Morales‐Briones et al., 2021), *Gomphrena* has previously been shown to be polyphyletic (Sánchez‐Del Pino et al., 2009; Bena et al., 2017). The placement of Achyranthoids largely aligns with previous studies by Di Vincenzo et al. (2018, 2025) and Ogundipe and Chase (2009). Only the monotypic genus *Arthraerua* (Kuntze) Schinz, here recovered as a sister to *Achyranthes bidentata* Blume, *Nototrichium humile* Hillebr., *Nelsia quadrangula*, and *Pandiaka involucrata* (Moq.) B. D. Jacks. (Figure 3), was in a polytomy with *Calicorema* Hook. f. in the backbone of Achyranthoids in Di Vincenzo et al. (2018). Within the Aervoids, *Ouret lanata* (syn. *Aerva lanata* (L.) Juss. ex Schult.) and *Aerva javanica* were recovered as sister species, contrary to the findings of Hammer et al. (2019), who found *Aerva javanica* as sister to *Paraerva* T. Hammer and *Ouret* Adans. as sister to *Ptilotus*.

The topology of the Amaranthoids is consistent with previous studies, with the largest genus, *Amaranthus*, being monophyletic (Di Vincenzo et al., 2018; Waselkov et al., 2018; Xu et al., 2024). Only the genus *Neocentema* has never been phylogenetically analyzed. Morphologically, it was placed in the subtribe Achyranthinae by Schinz (1934) but was later revised by Suessenguth (1949), who placed the genus in the Amaranthinae due to the root tip similarities of the embryo and suggested a close relationship with *Digera* Forssk. Here, we provide the first molecular confirmation of this morphological classification. Within the Celosioids, a sister relationship of the genera *Celosia* L. and *Hermbstaedtia* Rchb. was found, contrary to the Sanger‐based topology in Di Vincenzo et al. (2018), where *Celosia* and *Deeringia* were retrieved as sister genera, albeit not well supported. Although Celosioids comprise important crop plants like *Celosia argentea* L. (Ayodele, 2023), they are still severely understudied phylogenetically, and further molecular analyses are needed to resolve their evolutionary history.

### Conclusions

Our newly developed bait kit, Amaranthaceae1000, successfully targeted the intended loci. With a customized pipeline, we generated a well‐resolved phylogeny of Amaranthaceae s.s. While the phylogeny itself suffers from incomplete taxon sampling, it provides valuable insights, including the placement of previously recalcitrant groups, such as *Bosea* and *Charpentiera*, or previously non‐sampled genera, such as *Neocentema*. While further sampling is needed to address clade‐specific questions, we here illustrate that the Amaranthaceae1000 bait kit is a powerful and promising tool to address questions beyond pure phylogenomics, including spatio‐temporal reconstructions, population and comparative genomics, and trait evolution.

## AUTHOR CONTRIBUTIONS

D.F.M.B., T.K., A.Z.C., and G.K. designed the research. T.K. conducted the sampling in M and MSB, while A.Z.C. carried out the sampling in PERTH and CANB. T.K. performed the laboratory work. D.F.M.B. and T.K. analyzed the data. G.K. provided the funding. All authors interpreted the results. T.K. led the writing under the supervision of D.F.M.B., A.Z.C., and G.K., and all authors contributed to and approved the final version of the manuscript.

## Supporting information


**Appendix S1.** Loci extraction report from 29 Amaranthaceae s.s. transcriptomes using the CAPTUS pipeline. The completeness of recovered loci is color‐coded in a gradient from black (0%) to red (100%). (Top) Locus extraction using a target file containing only sequences of clade 1 (Gomphrenoids, Achyranthoids, and Aervoids) in blue. (Bottom) Locus extraction using a target file containing only sequences of clade 2 (Amaranthoids and Celosioids) in yellow.

**Appendix S2.** Fast‐Plast results showing the percentage of known angiosperm chloroplast genes recovered in 24 samples sequenced with the Amaranthaceae1000 baits.

**Appendix S3.** Astral‐Pro3 phylogenetic inference with all cleaned homologous trees from 57 transcriptomes and 24 samples sequenced with the Amaranthaceae1000 baits. The tree is rooted on members of the Caryophyllales, and support values on the branches correspond to local posterior probabilities (LPPs).

**Appendix S4.** Gene duplication mapping results. (Left) Histogram showing the percentage of gene duplications per branch. (Right) Phylogenetic inference from 24 species using ASTRAL IV with “monophyletic outgroup” (MO) ortholog trees, rooted on members of the Caryophyllales. Branch values indicate the proportion of duplicated genes with above 6 in bold. The stars mark the known WGD from Yang et al. (2018).

**Appendix S5.** Summary statistics of sequencing success obtained from HybPiper2. Metrics include species name, clade, number of reads, mapped reads, percentage mapped to targets, number of mapped genes, and additional gene recovery statistics (e.g., genes with contigs, sequences, or specific coverage thresholds). The table also reports paralog warnings (by length and depth), genes without or with stitched contigs, skipped contigs, chimera warnings, and the total bases recovered.

## Data Availability

All generated data are available from the National Center for Biotechnology Information (NCBI; Bioproject PRJNA1209683). The workflow for the bait design is available on Github (https://github.com/tinakiedaisch/bait_design_from_orthologs), and the bait design files are available on Dryad (https://doi.org/10.5061/dryad.k3j9kd5m6; Kiedaisch et al., 2025).

## Appendix

**Table:** Appendix 1. Voucher information for the newly sequenced Amaranthaceae s.s. species.

Species	Collector and collection no.	Collection date^a^	Collection locality	Herbarium barcode	DNA ID	Laboratory accession no.	SRA accession no.
*Aerva javanica* (Burm. f.) Juss. ex Schult.	D. Podlech 36191	02.10.1981	Yemen	MSB‐122362	4753	A9	SAMN48338600
*Alternanthera bettzickiana* (Regel) G. Nicholson	G.T. Prance et al. 15574	23.10.1971	Brazil	M‐0342909	4765	A16	SAMN48338601
*Amaranthus albus* L.	Richard R. Halse 8987	11.09.2013	USA	MJG015317	4729	A32	SAMN48338602
*Amaranthus macrocarpus* Benth.	SJ 9245	01.18.2005	Australia	—	840	A49	SAMN48338603
*Arthraerua leubnitziae* (Kuntze) Schinz	S.W. Breckle 10308	14.12.1986	Namibia	M‐0331726	4719	A25	SAMN48338604
*Bosea yervamora* L.	W. Hdhg. DT 2294	08.03.1995	Spain	M‐0331728	4730	A35	SAMN48338605
*Celosia elegantissima* Hauman	Reekmanns 10648	12.06.1981	Burundi	MSB‐122608	4731	A36	SAMN48338606
*Charpentiera elliptica* (Hillebr.) A. Heller	Paul C. Hutchison & John Obata 2824	15.01.1967	Hawaii, USA	M‐0331740	4757	A12	SAMN48338607
*Cyphocarpa angustifolia* Lopr.	K. Balkwill et al. 5344	13.01.1990	South Africa	M‐0331747	4720	A26	SAMN48338608
*Deeringia amaranthoides* (Lam.) Merr.	H. Y. Liang 63925	10.1933	China	M‐0331749	4767	A18	SAMN48338609
*Digera muricata* (L.) Mart.	Thulin 11452	2006	Oman	—	3731	A50	SAMN48338610
*Gomphrena arida* J. Palmer	A. Fraser 348	13.03.2001	Australia	CANB 636242.1	4734	A37	SAMN48338611
*Hebanthe erianthos* (Poir.) Pedersen	Strang 1057	23.07.1967	Brazil	M‐0335298	4768	A19	SAMN48338612
*Hermbstaedtia glauca* (J. C. Wendl.) Rchb. ex Steud.	H. Müller 772	01.08.1977	Namibia	M‐0331756	4763	A15	SAMN48338613
*Neocentema robecchii* (Lopr.) Schinz	Kazmi et al. 58	21.12.1977	Somalia	M‐0331769	4745	A8	SAMN48338614
*Nototrichium humile* Hillebr.	Otto Degener 20640	07.05.1950	Hawaii, USA	M‐0335291	4771	A20	SAMN48338615
*Ouret lanata* (L.) Kuntze	I. Hageman & R. Claßen 3224	01.10.1986	Kenya	MJG012475	4725	A30	SAMN48338616
*Pandiaka involucrata* (Moq.) B. D. Jacks.	R. Bartha 1/12	1967	Nigeria	M‐0335293	4726	A31	SAMN48338617
*Pfaffia glabrata* Mart.	A.P. Duarte 8305	19.07.1964	Brazil	M‐0335294	4772	A33	SAMN48338618
*Pleuropterantha revoilii* Franch.	J.J.F.E. De Wilde 5953	30.06.1969	Ethiopia	M‐0335302	4774	A21	SAMN48338619
*Ptilotus mollis* Benl	J. Hruban & D. Coultas SBJ‐01	10.02.2022	Australia	PERTH 09468528	4750	A4	SAMN48338620
*Ptilotus sericostachyus* (Nees) F. Muell.	PAGL 1/34	16.10.2005	Australia	PERTH 07508271	4751	A43	SAMN48338621
*Quaternella glabratoides* (Suess.) Pedersen	Pereira 7917	1963	Brazil	M‐0335295	4752	A44	SAMN48338622
*Sericorema remotiflora* (Hook.) Lopr.	Venter S. 12878	30.03.1988	South Africa	M‐0335307	4739	A5	SAMN48338623

*Note*: SRA = Sequence Read Archive.

^a^ Collection date is formatted as day.month.year.
