# Supplementary material for: Advancing phylogenomics in Amaranthaceae sensu stricto: Development and application of a new nuclear target enrichment bait set
Source: Appl Plant Sci. 2025 Aug 13;13(5):e70019. doi: 10.1002/aps3.70019 (PMC12542812; doi:10.1002/aps3.70019)

DOI 10.1002/aps3.70019

Appendix S1. Loci extraction report from 29 Amaranthaceae s.s. transcriptomes using the CAPTUS pipeline. The completeness of recovered loci is color-coded in a gradient from black (0%) to red (100%). (Top) Locus extraction using a target file containing only sequences of clade 1 (Gomphrenoids, Achyranthoids, and Aervoids) in blue. (Bottom) Locus extraction using a target file containing only sequences of clade 2 (Amaranthoids and Celosioids) in yellow.

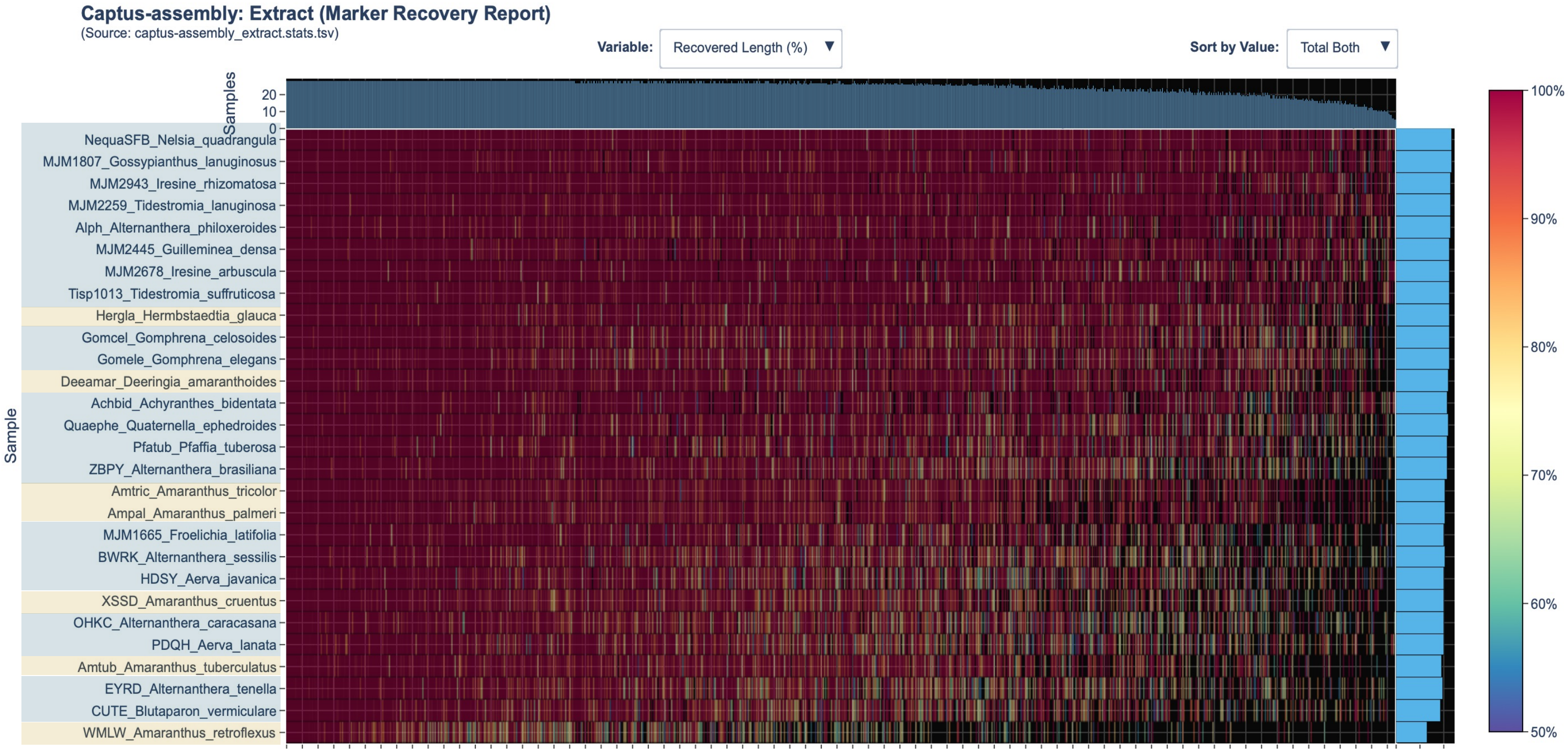

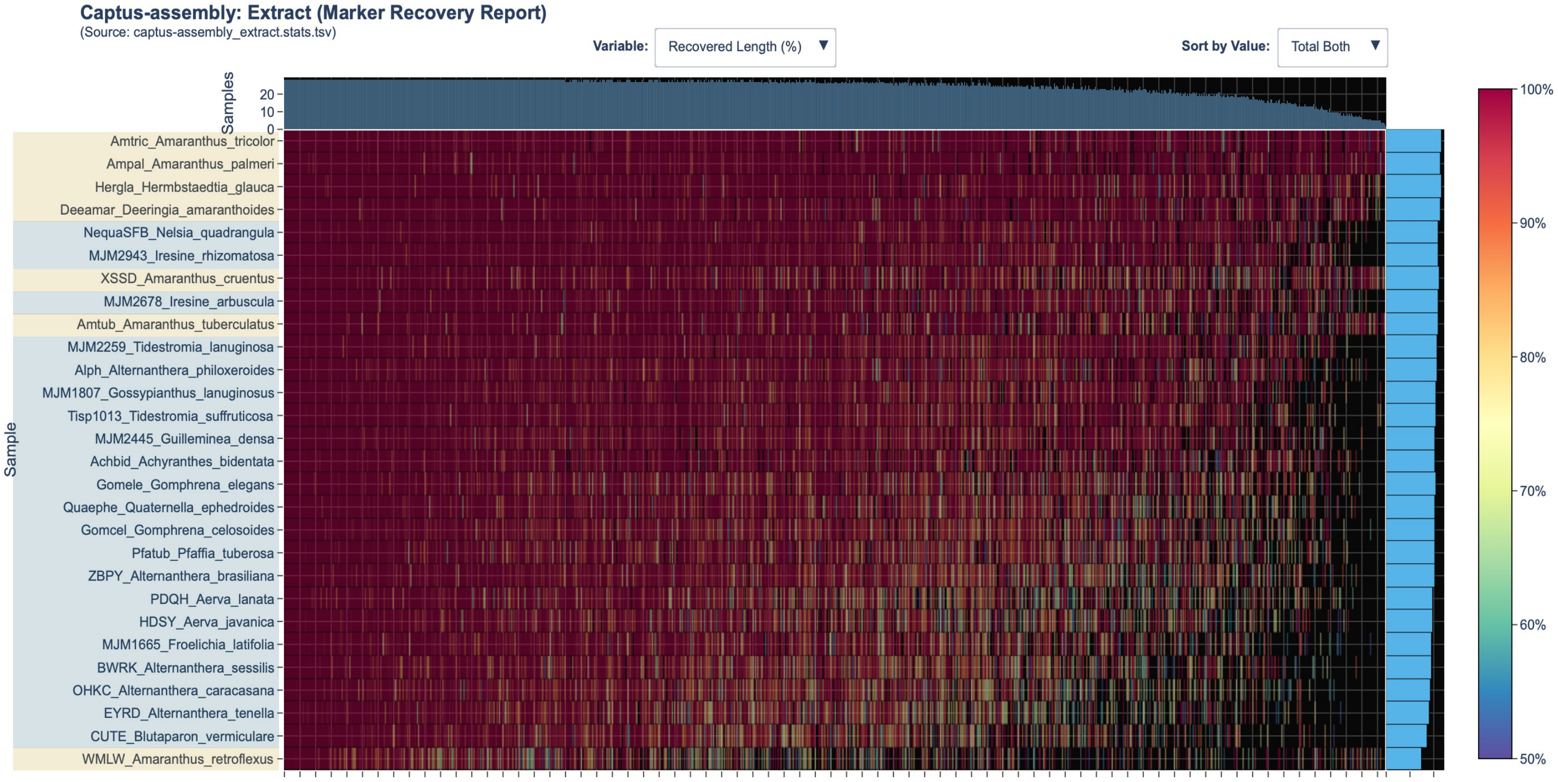

Supplement: Supplementary file 1 — Appendix S1. Loci extraction report from 29 Amaranthaceae s.s. transcriptomes using the CAPTUS pipeline. The completeness of recovered loci is color‐coded in a gradient from black (0%) to red (100%). (Top) Locus extraction using a target file containing only sequences of clade 1 (Gomphrenoids, Achyranthoids, and Aervoids) in blue. (Bottom) Locus extraction using a target file containing only sequences of clade 2 (Amaranthoids and Celosioids) in yellow. [file APS3-13-e70019-s005.pdf]
