# Supplementary material for: Advancing phylogenomics in Amaranthaceae sensu stricto: Development and application of a new nuclear target enrichment bait set
Source: Appl Plant Sci. 2025 Aug 13;13(5):e70019. doi: 10.1002/aps3.70019 (PMC12542812; doi:10.1002/aps3.70019)
Supplement: Supplementary file 2 — Appendix S2. Fast‐Plast results showing the percentage of known angiosperm chloroplast genes recovered in 24 samples sequenced with the Amaranthaceae1000 baits. [file APS3-13-e70019-s004.pdf]

DOI 10.1002/aps3.70019

Appendix S2. Fast-Plast results showing the percentage of known angiosperm chloroplast genes recovered in 24 samples sequenced with the Amaranthaceae1000 baits.

**Sample name**	**Percentage of known angiosperm chloroplast genes recovered**
Aerva javanica	17.28%
Alternanthera bettzickiana	66.67%
Amaranthus albus	59.26%
Amaranthus macrocarpus	79.01%
Arthraerua leubnitziae	3.70%
Bosea yervamora	6.17%
Celosia elegantissima	4.94%
Charpentiera elliptica	6.17%
Cyphocarpa angustifolia	37.04%
Deeringia amaranthoides	4.94%
Digera muricata	74.07%
Gomphrena arida	80.25%
Hebanthe erianthos	29.63%
Hermbstaedtia glauca	79.01%
Neocentema robecchii	24.69%
Nototrichium humile	4.94%
Ouret lanata	44.44%
Pandiaka involucrata	25.93%
Pfaffia glabrata	51.85%
Pleuropterantha revoilii	71.60%
Ptilotus mollis	82.72%
Ptilotus sericostachyus	45.68%
Quaternella glabratoides	79.01%
Sericorema remotiflora	62.96%
