# Supplementary material for: Advancing phylogenomics in Amaranthaceae sensu stricto: Development and application of a new nuclear target enrichment bait set
Source: Appl Plant Sci. 2025 Aug 13;13(5):e70019. doi: 10.1002/aps3.70019 (PMC12542812; doi:10.1002/aps3.70019)

DOI 10.1002/aps3.70019

Appendix S3. Astral-Pro3 phylogenetic inference with all cleaned homologous trees from 57 transcriptomes and 24 samples sequenced with the Amaranthaceae1000 baits. The tree is rooted on members of the Caryophyllales, and support values on the branches correspond to local posterior probabilities (LPPs).

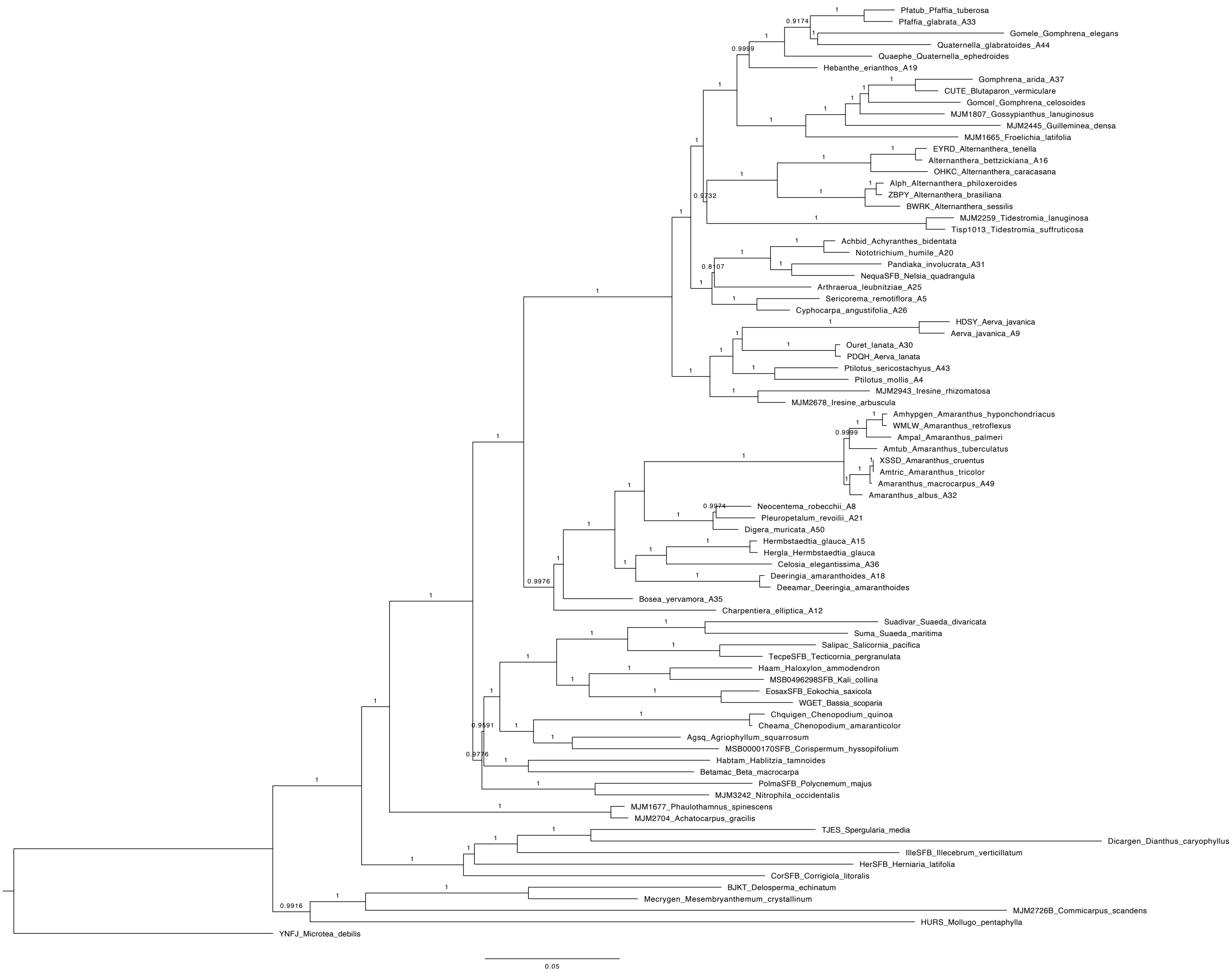

Supplement: Supplementary file 3 — Appendix S3. Astral‐Pro3 phylogenetic inference with all cleaned homologous trees from 57 transcriptomes and 24 samples sequenced with the Amaranthaceae1000 baits. The tree is rooted on members of the Caryophyllales, and support values on the branches correspond to local posterior probabilities (LPPs). [file APS3-13-e70019-s002.pdf]
