# Supplementary material for: Advancing phylogenomics in Amaranthaceae sensu stricto: Development and application of a new nuclear target enrichment bait set
Source: Appl Plant Sci. 2025 Aug 13;13(5):e70019. doi: 10.1002/aps3.70019 (PMC12542812; doi:10.1002/aps3.70019)

DOI 10.1002/aps3.70019

Appendix S4. Gene duplication mapping results. (Left) Histogram showing the percentage of gene duplications per branch. (Right) Phylogenetic inference from 24 species using ASTRAL IV with "monophyletic outgroup" (MO) ortholog trees, rooted on members of the Caryophyllales. Branch values indicate the proportion of duplicated genes with above 6 in bold. The stars mark the known WGD from Yang et al. (2018).

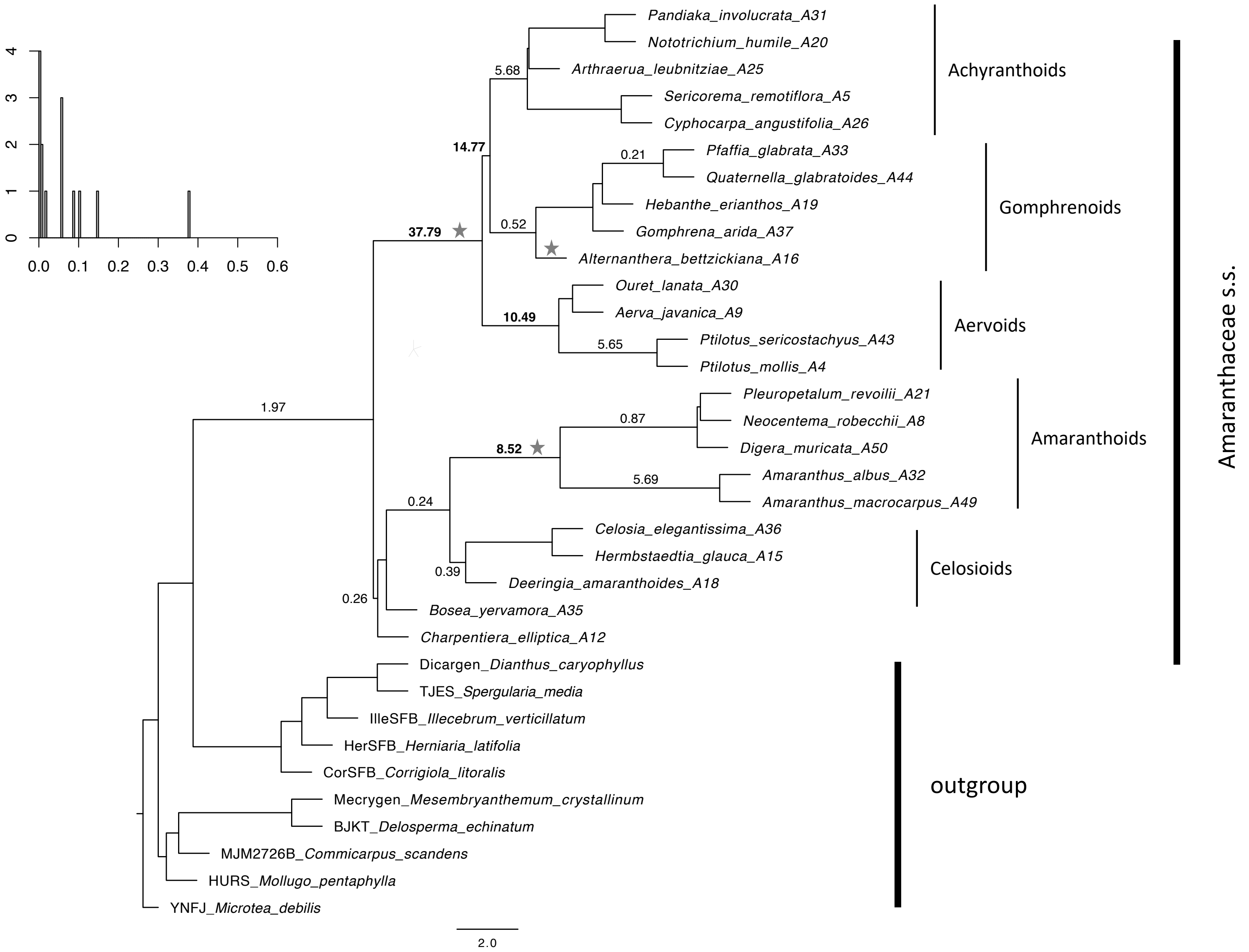

Supplement: Supplementary file 4 — Appendix S4. Gene duplication mapping results. (Left) Histogram showing the percentage of gene duplications per branch. (Right) Phylogenetic inference from 24 species using ASTRAL IV with “monophyletic outgroup” (MO) ortholog trees, rooted on members of the Caryophyllales. Branch values indicate the proportion of duplicated genes with above 6 in bold. The stars mark the known WGD from Yang et al. (2018). [file APS3-13-e70019-s003.pdf]
